# Assessing the role of bronchoscopy in the management of patients with acute leukemia—a transversal study and proposal of evaluation

**DOI:** 10.1016/j.htct.2025.103741

**Published:** 2025-02-19

**Authors:** José Vanildo Rodrigues de Oliveira, Carlos Wilson de Alencar Cano, José Vivaldo Moreira Feitosa Júnior, Guilherme Carneiro Barreto, Fernanda Rodrigues Mendes, Raphael Costa Bandeira de Melo, Elvira Deolinda Rodrigues Pereira Velloso, Vanderson Rocha, Eduardo Magalhães Rego, Wellington Fernandes da Silva

**Affiliations:** a Instituto do Câncer do Estado de São Paulo, Hospital das Clínicas da Faculdade de Medicina da Universidade de São Paulo, São Paulo 01246-000, Brazil; b Laboratory of Medical Investigation in Pathogenesis and Directed Therapy in Onco-Immuno-Hematology (LIM-31), Division of Hematology and Cell Therapy, Hospital das Clínicas da Faculdade de Medicina da Universidade de São Paulo, São Paulo 01246-000, Brazil

**Keywords:** Acute leukemia, Immunocompromised patients, Bronchoscopy, Infectious complications, Medical management

## Abstract

**Introduction:**

Bronchoscopy is frequently performed in the management of patients with acute leukemia due to their high susceptibility to infections. In this setting, it is performed in the context of lung infiltrates on imaging and persistent fever in immunocompromised subjects. This study aimed to evaluate the utility of bronchoscopy in patients with acute leukemia, its diagnostic yield, and its impact on management decisions.

**Methods:**

This is a single-center cross-sectional study that included patients diagnosed with acute leukemia of any phenotype who received intensive chemotherapy. Consecutive patients who underwent bronchoscopy as part of a work-up for associated infections were selected, while patients who had undergone bone marrow transplant were excluded. This study investigated patient characteristics and the impact of bronchoscopy on changes in clinical management.

**Results:**

Seventy-nine patients who underwent bronchoscopy at various stages of treatment were analyzed. The most frequent type of acute leukemia was acute myeloid leukemia, accounting for 68.3 % of cases. The induction phase was the most prevalent (29.1 %) treatment stage. Bacterial cultures were positive in 17 out of the 74 patients evaluated, with *Pseudomonas aeruginosa* being the most frequently identified microorganism*.* A change in medical management was observed in 18.2 % of cases, and only six patients experienced secondary complications.

**Conclusions:**

This is the first Brazilian study to evaluate the utility of bronchoscopy in managing infectious complications in patients with acute leukemia. The impact of bronchoscopy on clinical management was less than anticipated, largely due to its low yield in identifying causative agents. Nevertheless, it remains a safe procedure and can be useful in specific situations.

## Introduction

Patients with acute leukemia frequently face infectious complications at presentation or during intensive treatment. These infections are mainly of the respiratory tract, followed by bloodstream and urinary tract infections.^1^ In these patients there is an increased incidence of opportunistic infections due to basic changes in the immune system, which lead to a loss of cellular defense against pathogens.^2^ Patients with acute myeloid leukemia (AML) have qualitative and quantitative deficits in granulocytes and exhibit a reduced number of natural killer cells, which are important elements for innate immunity.^3^ Patients with acute lymphoblastic leukemia (ALL) have qualitative deficits in lymphocytes, resulting in hypogammaglobulinemia and reduced cell-mediated immunity, predisposing them to bacterial, viral and fungal infections.^4^

In patients diagnosed with acute leukemia, whether they are experiencing active disease or are in remission, the presence of febrile neutropenia, particularly when accompanied by respiratory symptoms, should trigger a recommendation for a computed tomography scan of the chest. This approach aims to promptly identify lung infiltrates and lesions suggestive of invasive fungal disease.^5^ Considering the number of potential microbiological causes and the potential toxicity associated with empirical therapy, particularly with antifungal and antimicrobial drugs, isolating the specific pathogens responsible is considered the ideal to manage patients.^6^

Bronchoscopy is frequently performed in neutropenic patients with hematologic malignancies who present lung infiltrates or persistent fever.^7^ Due to associated thrombocytopenia and risk of bleeding, there may be concerns about performing such invasive tests for etiological diagnoses.^8^ There are few clinical studies demonstrating the utility and safety of bronchoscopy in the investigation of infectious complications during the treatment of patients with acute leukemias.

Despite its feasibility, the role of bronchoscopy as a diagnostic method for the investigation of infections in immunosuppressed patients remains uncertain, with variable results in terms of diagnostic accuracy, and limited impact on management changes in some cases. Furthermore, it is important to emphasize that the diagnostic yield of this exam may be altered by the time the test was performed and by early use of antibiotics.^9^ In this study, we aimed to examine the diagnostic accuracy of bronchoscopy and the risk of procedural complications during the treatment of patients with acute leukemias.

## Materials and methods

### Study design

This study is a single-center retrospective transversal study, conducted at the hematology department at the Institute of Cancer of São Paulo (ICESP), University of São Paulo (USP), in Brazil. Clinical data were obtained from the databases of electronic medical records and managed using REDCap electronic data capture tools hosted at the University of Sao Paulo.^10^ The local ethics committee approved this study.

### Patients

Over 18-year-old patients diagnosed with acute leukemia of any phenotype, who underwent intensive chemotherapy between 2012 and 2022, were included. The intensive treatment regimens have been reported elsewhere, but, briefly, patients diagnosed with acute promyelocytic leukemia (APL) were treated with all-trans retinoic acid and intensive chemotherapy^11^, while ALL and AML patients received chemotherapy upfront.^12^^,^^13^ At our center, the local protocol recommended antifungal prophylaxis with fluconazole (Diflucan) during induction for AML and APL, anidulafungin for ALL and voriconazole for refractory and relapsed AML. The center does not routinely use antibiotic prophylaxis during induction.

Consecutive patients who underwent bronchoscopy to investigate an infectious focus at any stage of treatment were included. Subjects who underwent diagnostic bronchoscopy related to allogeneic bone marrow transplantation (BMT) or before the diagnosis of acute leukemia were excluded from this analysis.

## Microbiological tests

Whenever possible, direct microscopy and cultures were performed for each patient to investigate bacterial, fungal, and mycobacterial infections. Furthermore, testing for tuberculosis, cytomegalovirus (CMV) and pneumocystis was carried out by polymerase chain reaction (PCR). The galactomannan index in bronchoalveolar lavage was considered positive if equal to or greater than 0.5. ^14^

## Results

A total of 608 patients diagnosed with acute leukemia who received intensive chemotherapy were initially considered for this study. Of these, 79 (12.9 %) underwent bronchoscopy at some stage of treatment (except during the allogeneic BMT period) and were considered for analysis.

The most common subtype of leukemia was AML (54/79 patients; 68.3 %), followed by ALL (21/79 patients; 26.7 %), and APL (4/79 patients; 5 %). Regarding the treatment phase, 15 patients (19 %) performed bronchoscopy during pre-treatment, 23 patients (29.1 %) during remission induction, 19 patients (24.1 %) during consolidation, 14 patients (17.7 %) during salvage/reinduction, and eight patients (10.1 %) in remission.

Patients had a median age of 44 years (range: 18–71 years) and 47 patients (59.4 %) were male (Table 1). The most common reason for requesting the exam was investigation of lung infiltrate in 71 % of patients, followed by persistent fever in 29 %. Overall, 40.5 % patients were severely neutropenic (<0.5 g/L) on the day of the bronchoscopy, and had a median platelet count of 49×10^9^/L (interquartile range [IQR]: 30–103×10^9^/L). The procedure was performed while the patient was receiving antibiotic therapy in 76 patients (96.2 %), of which 62.0 % were also receiving antifungal treatment.

**Table 1:** Baseline characteristics.

Characteristic	Patients (*n* = 79)
Age - years median (range; IQR)	44 (18–71; 29.5–56.5)
Sex –n (%)	
Male	47 (59.4 %)
Female	32 (40.6 %)
Neutropenia (%)	40.5
Platelets (x 10^9^/L) (median, range, IQR)	42.0
Subtype of leukemia n (%)	
AML	54 (68.3 %)
APL	4 (5.0 %)
ALL or Mixed Phenotypic Leukemia	21 (26.7 %)
Treatment phase (%)	
Pre-treatment	19.0
Induction	29.1
Consolidation	24.1
Refractory/Retreatment	17.7
Remission	10.1

IQR: Interquartile range; AML: Acute myeloid leukemia; APL: Acute promyelocytic leukemia; ALL: Acute lymphoblastic leukemia.

Results of bronchoalveolar lavage analysis showed a positive bacterial culture in 17/74 patients (23 %) and positive fungal cultures in 7/71 patients (9.9 %). Screening for tuberculosis by PCR was positive in 3/60 patients, for CMV in 2/11 patients, and for *Pneumocystis jirovecii* in 1/8 patients.

The most common bacterial agent isolated was Pseudomonas aeruginosa in five patients, followed for Klebsiella pneumoniae in two patients. Infections by Enterococcus faecalis, Enterococcus faecium, Staphylococcus epidermidis, Staphylococcus haemolyticus, Acinetobacter baumanii complex, Stenotrophomonas maltophilia, Streptococcus oralis, Streptococcus viridans, and Streptococcus pneumoniae were also documented.

Regarding fungal agents, the most common was *Candida albicans* in three patients*. Candida krusei* and *Candida glabrata* were found in one patient each. Only one patient had a growth of *Mycobacteria tuberculosis* in the bronchoalveolar lavage. Galactomannan positivity occurred in 8/48 patients (16.6 %). Transbronchial biopsy was performed in only eight patients, with no isolation of microbiological disease in the histological assessment.

The procedure-related complication rate was low (only six patients) with three developing worsening of oxygenation, but without the need for tracheal intubation, two patients experiencing bleeding, and one patient evolving with pneumothorax (Table 2).

**Table 2:** Bronchoscopy outcomes and complications.

Changes in management	N (%)
Switching antimicrobial	5 (27.8)
Discontinuation antimicrobial	2 (11.2)
Starting antimicrobial or antifungal	7 (38.8)
Discontinuation antifungal	3 (16.7)
Keeping antimicrobial	1 (5.5)
Total	18
Bronchoscopy complications	
Hypoxia (desaturation)	3 (50.0)
Bleeding	2 (33.4)
Pneumothorax	1 (16.6)
Total	6

Bronchoscopy changed management in eighteen patients (22.78 %). The most common changes were the switching of antimicrobials (27.8 %) or initiating antimicrobial or antifungal therapy (38.8 %). Other procedures are described in Table 2. During the clinical follow-up, 24.4 % patients eventually died from pulmonary diseases.

## Discussion

There are few studies examining the role of bronchoscopy in the setting of acute leukemia, a disease with a particular profile of patients at high risk of opportunistic infection, and other complications due to profound cytopenias.^15^ In these patients, febrile neutropenia is an important cause of morbidity and mortality, despite the implementation of adequate management protocols.^16^ The accuracy of bronchoscopy in determining the etiologic diagnosis of lung infiltrates in leukemia patients is variable across the literature. The availability of molecular methodologies, local policies regarding prophylaxis, and local microbiology seem to strongly affect this heterogeneity.

In this study, bronchoscopy had a lower diagnostic yield than expected, with a low frequency of changes in the management of patients with acute leukemia. Some studies showed changes in patient management of around 30–40 %^17^, while others report lower percentages^25^, perhaps suggesting that the role of this procedure is limited.^18^

The treatment phase of acute leukemia in which bronchoscopy was most performed was induction (29.1 %), similar to other reports, this being the most critical treatment period, a time when treatment toxicities are added to the risks of the disease itself.^17^^,^^19^ Prior analysis from our group demonstrated that infection is the most common cause of death during induction in AML. Age, monocytic AML, adverse risk in the genetic stratification, Gram-negative colonization, and high levels of C-reactive protein are significant risk factors.^13^ The rate of neutropenic patients at the time of the examination was 40 %, with studies reporting rates from 19.4 to 94.0 % (Table 3).^20^

**Table 3:** Patient characteristics comparing different studies.

Paper citation	Total cohort (n)	Diagnosis of acute leukemia (%) and subtypes or other diseases	Median age in years (range, IQR)	Male sex n (%)	Severe Neutropenia <0.5*g*/L (%)	Platelet count (x 10^9^ cells/L)	Most common timepoint of undergoing of bronchoscopy
Von Eiff et al.^20^	103^a^	67 % - AML, ALL, MDS, blast phase of CML, ML and CLL	[16–83; -]	52 (57.7)	73.0	–	–
A Rabbat, Nature, Acute Leukemia 2009^a^^25^	73	100 % - AML	53 [-; -]	37 (50.6)	62.0	–	Induction (38.0 %)
Kim et al.^18^	187^b^	50,8 % - AML, ALL, Aplastic Anemia, LM and CLL	50 [-; 17–78]		19.4	–	–
Deotare et al.^19^	71	88 % - AML, ALL, Mixed Phenotypic Leukemia, MDS and Myelofibrosis	55 [46–63; -]	34 (48)	–	26	–
Susanne Ghandilli, Cancers, 2022	88	100 % - AML or ALL	59 [48–68; -]	59 (67)	94.0	–	Induction (58.0 %)
Present study	79	100 %	44 [29.5–56.5; 18–71]	47 (59.4)	40.5	49	Induction (29.1 %)

AML: Acute myeloid leukemia; ALL: Acute lymphoblastic leukemia; MDS: Myelodysplastic syndrome; LM: Lymphoid malignances; CLL: Chronic lymphocytic leukemia; CML: Chronic myeloid leukemia.

Data missing in articles.

a In this study, 103 bronchoscopies were performed, but only 90 patients were analyzed. Thirteen underwent to the procedure twice.

b In this study, AML and lymphoid malignances were included, but only the cohort of patients with AML are described and analyzed here.

Positivity of cultures, galactomannans and other microbiological tests was low, possibly because these tests had already been requested in the context of the empirical use of antifungals and antimicrobials, which are routinely introduced for febrile neutropenia. Among bacterial infections, *Pseudomonas aeruginosa* (five patients) was the most common isolated agent, reinforcing the idea that gram-negative pneumonia is more frequent in patients with prolonged hospitalization and persistent neutropenia (Table 4).^21^

**Table 4:** Microbiological results and treatment modifications comparing the studies.

Paper citation	Bacterial infection results (%)	Most common bacterial infections	Most common fungal infections	Galactomannan positivity (%)	Treatment modifications (%)
Von Eiff et al.^20^	64 % (only pathogenic bacteria)	*Legionella pneumophila* (1st)/*Pseudomonas aeruginosa* (2nd)	*Candida species* (1st)/ *Aspergillus species* (2nd)	–	37.7 %
A Rabbat, Nature, Acute Leukemia 2009^25^	13.2 %	*Stenotrophomonas maltophila* (1st)	*Candida/Aspergillus/* *Trichosprium* (1st)	–	17 %
Kim et al.^18^	41.8 %	*Acinetobacter sp*/MRSA (1st) *Pseudomonas* (2nd)	*Pneumocystis jirovecii* (1st) *Candida* (2nd)	31.5 %	30.1 %
Deotare et al.^19^	11.2 %	*Pseudomonas aeruginosa/* Klebsiella pneumoniae (1st)	*Pneumocystis jirovecii/ Candida* (1st)	–	45 %
Susanne Ghandilli, Cancers, 2022	6 % (only pathogenic bacteria)	*Stenotrophomonas maltophila* (1st)	*Aspergillus fumigattus* (1st)	–	43 %
Present study	21.5 %	*Pseudomonas aeruginosa* (1st)	*Candida albicans* (1st)^a^	16.6 %	22.8 %

Data missing in the articles.

a In the current study, positivity samples for Candida were found in five bronchoscopies, but yeasts could correspond to a non-pathogenic germ.

Documented fungal infections were scarce, with only five cases, the highest prevalence being *Candida albicans*, followed by azole-resistant fungi, such as *Candida krusei* and *Candida glabrata*. The presence of yeasts in a bronchoscopy could suggest only contamination, without affecting the clinical outcome. However, the low prevalence may reflect the appropriate role of prophylaxis for candidemia and filamentous fungi.^22^^,^^23^

The complications related to the bronchoscopy are variable, ranging from tachycardias, bradycardias and cough to severe bleeding, cardiac arrhythmia requiring treatment, seizures, myocardial infarction, pulmonary edema, hypotension requiring vasopressors, pneumothorax and death.^24^ In the present study, the complication rate for the procedure was 7.5 % (six patients), in particular hypoxemia, consistent with findings from other studies (Table 4).^19^ No patient in this study died as a result of undergoing this exam. Therefore, this low rate seems to show that the procedure is safe despite the context in which this patient population is inserted.

Some limitations of this study are the retrospective single-center design, as well as the small number of patients (*n* = 79). However, this was a representative sample of consecutive patients. Possible matters related to the procedure were logistical issues, such as inaccuracy in ordering tests or unavailability of all molecular and microbiological tests. Regarding the yield of this exam, it probably was affected by the initiation of antibiotics and antifungals prior to the procedure, which may have led to false negative results.

In summary, after reviewing the data of this study and available literature on this matter, we propose an algorithm for evaluating lung infiltrates in acute leukemia patients, as well as the diagnostic methods that can be used for etiological purposes (Table 5).

**Table 5:** Tests and procedures for a patient with acute leukemia and lung infiltrates (adapted from British Thoracic Society guidelines).

Reasons to perform the bronchoscopy
Persistent fever in patients with neutropenia (more than seven days) despite of the use of empiric broad-spectrum antibiotics and chest CT scan showing new pulmonary infiltrates
Caution before the procedure
Perform coagulation studies, platelet count and hemoglobin concentration. For BAL, a platelet count above 20×10^9^ is recommended. Consider platelet transfusion if a transbronchial biopsy is needed, keeping a platelet count of at least 50×10^9^. The risk of biopsy needs to be weighed against the potential for benefit. Discontinuation of anticoagulants. Discontinuation of clopidogrel (seven days prior to procedure). Low-dose aspirin alone can be continued.
Possible serious adverse events
Cardiac arrythmia Seizures Myocardial infarction Pulmonary edema Pneumothorax requiring aspiration/intercostal drain. Respiratory failure Death
Necessary Tests
Microscopic analysis Direct fungal analysis Cytomegalovirus PCR Tuberculosis PCR Pneumocystis PCR Cultures for bacteria, fungal and mycobacteria BAL Galactomannan
Additional Tests
Herpes Simples Virus PCR Influenza A and B PCR Rhinovirus PCR Adenoviruses, coronaviruses PCR

CT: Computerized tomographic; BAL: Bronchoalveolar lavage; PCR: Polymerase chain reaction.

## Conclusion

The role of bronchoscopy in the investigation and treatment of patients with acute leukemia remains controversial due to its lower-than-expected diagnostic yield and the low rates of change it provokes in medical management. Nevertheless, it is important to recognize the value of this exam in identifying specific infections like tuberculosis, which can be challenging to diagnose using other methods.

Furthermore, the safety of this procedure in this clinical context supports the recommendation of performing it as early as possible when pulmonary infiltrates are detected because the initiation of antimicrobial therapies can affect results. However, until now, the real impact on survival of these patients still remains undefined and perhaps the bronchoscopy can be omitted in some patients without prejudicing treatment.

## Conflicts of interest

None.
